# Intramedullary Nailing in Femoral Diaphyseal Fractures: A Retrospective Multicenter Cohort Study

**DOI:** 10.3390/life15040540

**Published:** 2025-03-26

**Authors:** Fábio Lucas Rodrigues, Ana Lya Moya Ferrari, Fernando Ferraz Faria, Rafael Luiz Emmanoel Pinto, Manuela Fernandes Lopes, Maria Eduarda Alencar Santos, Evelyn Cardenas Varela, Manuel Jucelino Lopes Filho, Marianna Nogueira Cecyn, Nelson Henrique Carvalho de Oliveira

**Affiliations:** 1 Orthopedics and Traumatology Residency Program, ABC Medical School, Santo André 09060-870, Brazil; 2 Research and Development Department, Biomecanica, Jaú 17212-811, Brazil; analya_mf@hotmail.com; 3 Orthopedics and Traumatology Residency Program, Cajuru University Hospital, Curitiba 80050-350, Brazil; ferrazfaria@hotmail.com (F.F.F.); prafaelluiz45@gmail.com (R.L.E.P.); 4 Orthopedics and Traumatology Residency Program, Adriano Jorge Hospital Foundation, Manaus 69079-015, Brazil; manuela_lopes92@hotmail.com (M.F.L.); meduarda.alencar.santos@gmail.com (M.E.A.S.); evelyncavarela0612@yahoo.com.br (E.C.V.); mjulf@hotmail.com (M.J.L.F.); nelsonhenrique.co@gmail.com (N.H.C.d.O.); 5 Paulista School of Medicine, Department of Psychobiology, Federal University of Sao Paulo, São Paulo 04724-000, Brazil; marianna.cecyn@unifesp.br

**Keywords:** intramedullary fracture fixation, consolidation, femoral fractures, diaphyseal, shaft, nail

## Abstract

Intramedullary nails (IMNs) are the most frequent surgical fixation method for femur fractures. Although IMNs provide good healing outcomes and low complication rates, concerns persist regarding potential complications such as malunion, nonunion, and infections. This multicenter retrospective study aims to assess the epidemiology and outcomes of IMNs for diaphyseal femoral fractures. Data from 91 patients who underwent IMN fixation at two Brazilian hospitals between 2020 and 2024 were analyzed, with a mean age of 33.3 years (SD ± 12.7) and 76.9%% of male patients. Traffic accidents were the most common mechanism of trauma (84.61%). The bone healing rate was 96.7% within six months, and 98.9% within one year, with a complication rate of 3.26%, including two cases of pseudoarthrosis, one case of pseudoarthrosis and infection, and two reoperations. There was a significant association between previous external fixation and fracture type (open/closed) (χ^2^(1) = 17.5, p_Fischer_ < 0.001). Previous external fixation was also associated with lower consolidation rates six months post-surgery (χ^2^(1) = 9.83, p_Fischer_ = 0.031), but not after one year (χ^2^(1) = 8.19, p_Fischer_ = 0.11). The retrograde approach was associated with a lower consolidation rate after six months (χ^2^(1) = 6.98, p_Fischer_ = 0.027), but no significant association was found after one year (χ^2^(1) = 2.27, p_Fischer_ = 0.308). Only one patient with pseudoarthrosis did not consolidate after one year. The outcomes support the efficacy of IMNs in achieving bone consolidation with low complication rates.

## 1. Introduction

Femoral fracture numbers have risen disproportionately with population growth [1]. Between 1998 and 2021, there was a rise of 71% in the incidence of femoral diaphyseal fractures among males and a 100% increase among the female population [2]. Estimates suggest that femoral fractures occur at rates of 9.5 to 19 per 100,000 individuals per year, rising to 19.9 per 100,000 in the elderly population [3]. These fractures increased the mortality in injured older adults by 58.5%, especially when combined with additional risk factors such as diabetes mellitus, open fractures, osteoporosis, smoking, poverty, and lower household income [4]. While grounded-level falls are primarily associated with older adults [5], traffic accidents and falls from heights are the most common causes of femoral fractures among younger populations [6,7]. The treatment of femoral fractures can be associated with multiple factors to potential failure, including demographic factors such as age, gender, ethnicity, and socioeconomic status, all of which affect bone healing [3]. Therefore, even though fracture healing failure can be multifactorial, providing a stable fixation to enhance the healing process remains crucial [8].

Intramedullary nails (IMNs) are widely used for the fixation of long bone fractures and are the principal method for femoral diaphyseal fracture fixation [9]. Among surgical fixation methods, IMNs are considered a minimally invasive treatment that preserves soft tissue and blood flow, providing good biomechanical performance [10]. A comparison between IMNs and locking compression plates for femur fractures has demonstrated that IMNs result in better outcomes, including lower rates of malalignment, claudication, and faster return to functional activities [11]. Femoral fractures presented complication rates of 4.9% [12], bone healing rates from 72% to 100% [13,14,15], and an average healing time of 5.8 months [15], with healing rates of 91,4% at 12 months [16]. As a less invasive method, IMNs offer a lower risk of in-hospital complications [17]. Despite significant advances in IMN technology, challenges still persist, such as achieving rotational stability, improving union rates, and reducing infection risks, particularly in patients with fractures caused by high-energy trauma and with multiple comorbidities [18]. Among the complications, the average nonunion rate for femoral diaphyseal fractures is 2.4%, ranging from 0 to 14% [19], while infection rates vary between 0.6% and 6.1% [20,21,22]. The analysis of risk factors for infections and nonunion is crucial to better understanding and establishing predictive factors for those complications. Perioperative blood transfusion, postoperative non-weight-bearing, delay to full weight-bearing smaller than 12 weeks, and fracture type are risks factors associated with femoral nonunion [7], while open fractures, diaphyseal fractures, and soft tissue injury are risk factors for infections [19].

In Brazil, the most common treatment for femoral diaphyseal fractures is an antegrade approach with reamed nailing [23]. However, apart from injury characteristics, the treatment depends on the availability of healthcare resources, equipment, and medical teams [23]. Recent studies of the Brazilian adult population have focused on the epidemiology of femoral fractures in older adults without specifically addressing treatment with IMNs [24,25,26,27]. Another study examined IMN outcomes, particularly infection rates in tibial and femoral fractures, but without exploring other complications [28]. No published investigation has addressed the epidemiology of IMN treatment for femoral diaphyseal fractures in the Brazilian adult population. Considering the factors related to potential failures and the variation in outcome rates across the literature, epidemiological studies of specific populations, such as the one proposed in our study, are important for guiding healthcare policies with accuracy regarding demographic data.

The objective of this multicenter retrospective study is to analyze the epidemiology of femoral diaphyseal fractures and evaluate the management and outcomes of IMN treatment in the Brazilian population in terms of consolidation and complications rates.

## 2. Materials and Methods

This retrospective multicenter cohort study investigated diaphyseal femoral fractures managed with intramedullary nailing (IMN). Data were collected from two Brazilian institutions, Cajuru University Hospital, a teaching hospital located in Curitiba, PR, and Adriano Jorge Hospital Foundation, a public hospital in Manaus, AM, between August 2020 and January 2024. In both institutions, the Intramedullary Nail Orion SP Femur or Intramedullary Nail SP2 Femur (Biomecanica, Jaú, SP, Brazil) type was used for fracture fixation. These hospitals were selected based on their history of using these specific devices.

The research was approved by the Ethics Committee of the ABC University Medical School (protocol number CAAE 82645524.7.1001.0082), according to the guidelines of the Brazilian National Council for Research Ethics. This study employed a retrospective methodology, collecting data from medical records without identifying individuals and reporting findings in aggregate form. No data that allowed patient identification were withdrawn from hospital systems. Aside from that, no therapeutic interventions were implemented. Therefore, the requirement for an informed consent form was waived.

### 2.1. Inclusion and Exclusion Criteria

Eligible participants included male and female patients aged 18 years or older at the time of surgery who underwent IMN for femoral diaphyseal fractures, with a minimum follow-up of one year. Patients with incomplete follow-up or those younger than 18 years at the time of surgery were excluded.

### 2.2. Data Collection and Statistical Analysis

The data collection procedures were made through an online form developed in Google Forms by the researcher responsible for data analysis. The form was completed by researchers from both institutions based on medical records. This approach ensured standardized data collection across the two institutions. The collected data were tabulated and reanalyzed by the researchers responsible for data analysis to ensure compliance with the study exclusion/inclusion criteria. Data collected included demographic information (gender, age, comorbidities, and history of substance use), fracture details (fracture type: open or closed, Müller-AO classification—assessed by blinded researchers independently from previous classifications, laterality, and trauma mechanism), and postoperative information, including reoperation, complications such as infection, malunion, material failure, or pseudoarthrosis, and consolidation. Bone consolidation was assessed six months and one year after surgery, according to hospital follow-up standard procedures, with radiographic imaging to confirm bone consolidation. Radiographic evaluation was carried out by an independent researcher who was not involved in the surgery. The evaluation considered the cortical continuity in at least three cortices (anteroposterior and lateral views). Additional clinical data assessed included initial treatment at another healthcare facility, multiple traumas, history of previous surgeries on the fracture, and use of external fixation.

Statistical analysis was conducted using Jamovi 2.4.11 (https://www.jamovi.org/, (accessed on 17 January 2025)). Categorical variables were presented as absolute (n) and relative (%) frequencies. Comparisons between categorical variables were performed using the chi-square and Fisher’s exact tests, with a 95% confidence level and a significance threshold of *p* < 0.05. Power analysis and real alpha were post hoc calculated for the exact Fischer test using G*Power (version 3.1.9.7). Participants with insufficient follow-up information (six months or one year) were excluded before the analysis.

## 3. Results

### 3.1. Patient Characteristics

**Figure 1:**
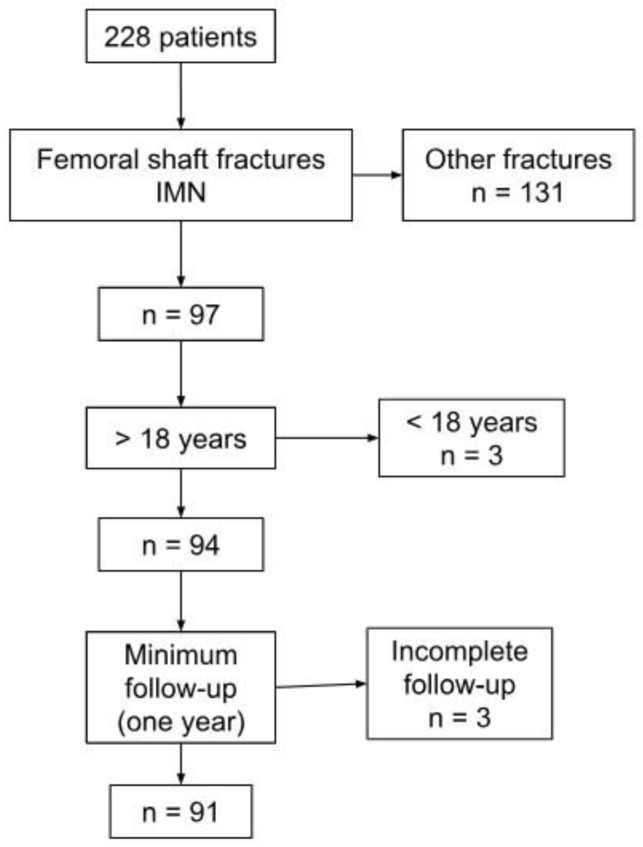
Patient inclusion/exclusion criteria.

During the data collection, 228 patients with lower limb fractures were treated with IMN at both institutions. Intramedullary nailing of femur diaphysis was performed in 97 patients. Of these, 94 patients met the inclusion criteria. However, 3 were excluded due to insufficient follow-up information (six months or one-year), leaving 91 patients for analysis. The definition of the final sample is presented in Figure 1.

The mean age of the included patients was 33.3 years (SD ± 12.7) (Table 1), and 76.9% were male. Sixteen patients (17.6%) reported abstaining from alcohol, tobacco, and illicit drugs. Most patients (82.4%) reported substance use, including thirty-nine alcohol users and three illicit drug users. Thirty-three patients reported the use of both alcohol and tobacco, while two reported using all three substances. No patient reported using only tobacco. The majority of patients (94.5%, n = 86) had no comorbidities. Among the five patients with comorbidities (5.5%), the conditions identified were systemic arterial hypertension in two patients (2.2%), systemic arterial hypertension combined with type II diabetes mellitus in one patient (1.1%), systemic arterial hypertension with prostate hyperplasia in another (1.1%), thyroid diseases in one patient (1.1%), and systemic arterial hypertension combined with both type II diabetes mellitus and thyroid diseases in one patient (1.1%).

### 3.2. Injury Characteristics

Traffic accidents were the most common mechanism of trauma (84.61%), followed by falls from height (5.49%), ground-level falls (3.29%), and sport-related trauma (3.29%) (Table 2). Two patients (2.19%) sustained femoral fractures due to gunshot wounds, while one patient (1.09%) fractured the femur from a horse kick. A non-significant trend was observed, with fractures on the left side in 56.0% of cases. The Müller-AO classification type 32A was the most common fracture (65.9%), followed by type 32B in 34.1% of cases. Most fractures (89.0%) were closed, and most patients (89.0%) had not undergone previous external fixation. Antegrade nailing was the most common fixation method (69.2%), while 30.8% underwent retrograde nailing. In 74 patients (81,31%), the treatment was initiated in another healthcare facility. Most patients (86.8%) had no associated fractures, and 89.0% had no injuries in other systems. The abdominal system was affected in 4.39% of the cases, while the thoracic system was affected in 2.19% of the patients. The information about trauma in another system was unavailable for four patients (4.39%). There was no history of fractures in the affected limb.

Only 2 patients (2.2%) required reoperation, and 88 patients (96.7%) achieved bone consolidation within six months (Table 3). After one year, 98.9% of the patients had achieved consolidation, including the two reoperated patients. The only comorbidity associated with complications was systemic arterial hypertension in the patient who developed pseudarthrosis and did not undergo reoperation, remaining the only non-consolidated case after one year in our cohort. Complications occurred in 3.3% of cases, with isolated pseudarthrosis (2.2%) and pseudarthrosis with infection (1.1%). All complications occurred in patients with closed fractures and retrograde fixation, and none of these patients were alcohol, tobacco, or drug users.

### 3.3. Subgroup Analysis

We found a significant association between previous external fixation and fracture type (χ^2^(1) = 17.5, p_Fischer_ < 0.001, Table 4). Among patients with closed fractures, 93.8% (n = 76) did not use an external fixator before IMN, and only five (6.2%) underwent previous external fixation. Among the patients with open fractures, 50.0% (n = 5) had previous external fixation, while five (50.0%) did not use external fixation. We found a significant association between previous external fixation and consolidation within six months (χ^2^(1) = 9.83, p_Fischer_ = 0.031, Table 5). Among patients who did not use an external fixator, only one (1.2%) did not consolidate within six months. Among patients who used an external fixator, eight patients (80.0%) consolidated within six months. There was no significant association between previous external fixation and consolidation within one year (χ^2^(1) = 8.19, p_Fischer_ = 0.11, Table 6). Only three patients did not consolidate within six months, one without external fixation and two with external fixation. After one year, the fractures of two reoperated patients were consolidated, one with prior external fixation and the other without, both with closed fractures. One of the patients with prior external fixation, in addition to pseudarthrosis, presented infection. The patient who did not consolidate the fracture within one year had pseudarthrosis and underwent retrograde nailing. We found a significant association between nailing approach and consolidation within six months (χ^2^(1) = 6.98, p_Fischer_ = 0.027, Table 7). All patients with anterograde nailing consolidated within six months (n = 63, 100%), while 89.3% (n = 25) of the patients with retrograde nailing consolidated the fracture six months after surgery. There was no significant association between the nailing approach and consolidation within one year (χ^2^(1) = 2.27, p_Fischer_ = 0.308, Table 8).

## 4. Discussion

This study retrospectively evaluated the epidemiological profile of IMN in femoral diaphyseal fractures, confirming global patterns in gender, age group, and trauma mechanism. We found high rates of consolidation and low reoperation and complication rates among the Brazilian population, with significant associations between previous external fixation and fracture type, and between previous external fixation and nailing approach with consolidations after six months.

The predominance of males (76.9%) is consistent with higher incidences of diaphyseal femoral fractures in the male population [3,9,12,21]. This trend is inverted in studies targeting the elderly population, with the dominance of female patients [29,30]. A similar tendency occurs in the elderly Brazilian population, where hospitalization due to femoral fractures among the female population is 1.7-fold higher compared to males [24]. The young sample of our study (mean 33.3 years) falls within the range reported in other studies [9,21], reinforcing the relationship between age and fracture location. Young patients (mean age 33.5 years) present femoral shaft fractures, whereas older patients (mean age 53.1 years) present a predominance of distal femoral fractures [5]. Previous studies in the Brazilian population also reinforce the pattern of the mechanism of trauma and age, with simple falls frequent in women above 60 years and car accidents with peak incidence among age groups between 20 and 30 years [31]. Femoral fractures usually present a bimodal distribution of high-energy (traffic accidents and falls from heights) trauma in young males [6,7,9] and low-energy trauma (ground-level falls) in elderly females [5,9]. Traffic accidents represent 76% of the causes of femoral fractures, while falls cause 15% of the injuries [9]. Our findings corroborate this trend, with traffic accidents as the leading cause of fractures (84.61%), followed by falls from heights and ground level (8.78%) in a younger cohort. Most patients in our cohort received initial treatment in another healthcare facility. Trauma victims transferred from emergency departments or secondary hospitals to tertiary hospitals to receive specialized treatment aged between 27 and 39.1 years and have traffic accidents as the predominant mechanism of trauma [32]. Those interhospital transfers are a regular practice in Brazilian hospitals, and patient characterization is aligned with our cohort.

Even with traffic accidents as the leading trauma mechanism in our sample, most patients (86.8%) had no associated fractures, while 89.0% had no trauma in other systems. These results contrast with previous reports associating traffic accidents with a higher prevalence of associated fractures [33]. The literature reports reoperation rates of 22% in femoral fractures caused by high-energy trauma [22]. Even with the low reoperation rate in our study (2.2%), the two reoperated fractures resulted from traffic accidents. Causes for reoperation in femoral fractures include nonunion (2.9%), delayed union (2.2%), deep infection (0.6%), mechanical failure (0.6%), and malunion (0.3%) [19]. In our sample, both patients developed pseudoarthrosis, and one of them, besides pseudoarthrosis, presented infection. Pseudoarthrosis is characterized by nonunion of the fracture, identified by the lack of radiographic consolidation and requiring revision surgery [34]. There is no register of revision surgery for the third patient with pseudarthrosis, the only fracture non-consolidated after one year in our cohort. The patient in question had systemic arterial hypertension, which can raise concerns for surgical procedures due to the increased risk of complications and in-hospital mortality [35,36]. However, data do not offer additional information, including noninvasive treatment. Therefore, explanations are only conjectures. In our study, we did not find an association between comorbidities and complications. However, an association of diabetes and hypertension with nonunion was observed in patients with femoral diaphyseal fractures [12]. Reduced osteoblast activity and increased osteoclastogenesis are mechanisms associated with hypertension, leading to bone loss and affecting the skeletal system [37]. Data on nonunion of femoral fractures varies in the literature, reaching 22% [7], with estimated incidences reported in 200 cases per million per year [18], or 13 per 1000 nonunions of pelvis and femur per year [38]. Besides anatomical factors, age was associated with the likelihood of nonunion, with the incidence of femur and pelvis nonunion decreasing as age increased [38]. Even though previous studies have reported an association between smoking history and nonunion rates [3], we did not find this association among our cohort. Although smoking is associated with nonunion, this risk is higher in active smokers compared to non-active smokers [39]. In our sample, 35 patients reported tobacco use, but our data did not include information on the frequency of use or whether the patients were active smokers at the time of surgery. For robust results in this sense, further investigations are needed.

In our sample, 11.0% of the fractures were previously treated with an external fixator, a method commonly used for damage control in unstable fractures, with patients transitioning to IMN when clinical conditions permit [40]. A previous study on femoral diaphyseal fractures reported no significant differences in union rates, healing outcomes, or complications between patients who received provisional external fixation and those who underwent direct fixation with IMN [21], and use of the previous external fixator was not associated with polytrauma or open fracture [7]. Nevertheless, our results showed a significant association between open fractures and external fixation and a significant association between external fixation and consolidation at six months. More patients in the subgroup treated directly with IMN achieved consolidation after six months than those previously treated with external fixation. However, this difference was not significant in the consolidation after one year. The external fixator is indicated for unstable fractures, which may be a factor influencing consolidation outcomes in our study and could be related with the association between external fixation and type of fractures observed in our results. Unstable femoral diaphyseal fractures are associated with young male victims of high-energy trauma, such as in our cohort and those fractures tend to have a longer consolidation time [31]. Moreover, external fixation is commonly used in open fractures, frequently presenting a commitment to surrounding soft tissue. Depending on the damage and the consequent classification of the fracture, there is an increased risk of complications that can lead to nonunion and affect bone consolidation [7,21,29,40]. Our data do not include the Gustilo–Anderson classification; however, this variable may be the principal cause for no consolidation observed in our findings. Further evidence is needed to address confounding variables and the relationship between previous external fixation and consolidation in IMN treatment. Infection is among these complications that can influence consolidation and expected in femoral fractures. Femoral shaft fractures presented lower rates (0.6%) of deep infection than distal femur fractures (2.4%) [19]. The low rates of deep infection, nonunion, malunion, claudication, and better outcomes in restoring functional activities contribute to the preference for IMN over plate fixation in femoral fractures [11]. The only infection in our sample occurred in a patient with a closed fracture who also developed pseudoarthrosis.

The literature reports comparable healing rates for antegrade and retrograde nailing (94.5% and 93.2%, respectively) [41]. Retrograde nailing was a significant risk factor for malunion and nonunion in femoral diaphyseal fractures [17,19]. Similarly, our findings revealed that all three patients with pseudoarthrosis underwent retrograde fixation, and the association between the nailing approach and consolidation at six months was significant, but after one year, there was no significant association between nailing approach and consolidation rates. No cases of malunion occurred in our sample.

The limitations of our study should be considered, and our findings should be carefully interpreted. Due to its retrospective multicenter design, some variables in surgical techniques, such as the fracture reduction methods, time between fracture and surgery and patient’s postoperative management, and detailed fracture characteristics were not accounted for in the analysis. Moreover, the study design did not include a control group as a comparison, which would provide stronger evidence for our findings. Nevertheless, due to the significant findings in our study, prospective studies are indicated to strengthen the evidence for the association between retrograde nailing and poor bone consolidation and offer explicit evidence for the association between external fixation and consolidation. Other variables such as vitamin D levels and Gustilo–Anderson classification should be considered in future investigations. Despite these limitations, our findings are consistent with the existing literature while addressing the ongoing discussion with recent data from Brazil and supporting evidence that IMN is a safe and efficient method for femoral diaphyseal fractures.

## 5. Conclusions

This study emphasizes the epidemiology and outcomes of IMN in femoral diaphyseal fractures within the Brazilian adult population. The primary injury mechanism of femoral diaphyseal fractures was high-energy trauma from traffic accidents, predominantly affecting young males, with prevalence of closed fractures. Open fractures were associated with previous external fixation, while previous external fixation and retrograde nailing were associated with non-consolidation within six months, but this association was not significant one year after surgery. Despite complications such as infection and pseudoarthrosis, their incidence was low, with high rates of consolidation. IMN should be considered as the primary treatment option for femoral diaphyseal fractures. However, clinicians should remain alert to potential complications, especially in cases involving previous external fixation and retrograde nailing during the first six months post-surgery.

## Figures and Tables

**Table 1:** Participant demographic data.

Sex	n (%)
Male	70 (76.9%)
Female	21 (23.1%)
Age	Mean (SD)
	33.3 ± 12.7
Substance use	n (%)
Yes	75 (82.4%)
No	16 (17.6%)
Type of substance (n = 75)	n (%)
Alcohol	39 (52.0%)
Tobacco	0 (0.0%)
Illicit drugs	1 (1.33%)
Alcohol and tobacco	33 (44.0%)
Alcohol, tobacco, and illicit drugs	2 (2.66%)

n = absolute frequencies; % = relative frequencies.

**Table 2:** Fracture characteristics, trauma mechanisms, associated injuries, and management of fracture.

Laterality	n (%)
Right	40 (44.0%)
Left	51 (56.0%)
Müller-AO classification	n (%)
32 A	60 (65.9%)
32 B	31 (34.1%)
Fracture type	n (%)
Open	10 (11.0%)
Closed	81 (89.0%)
Prior external fixation	n (%)
Yes	10 (11.0%)
No	81 (89.0%)
Associated fractures	n (%)
Yes	12 (13.2%)
No	79 (86.8%)
Traumas in other systems	n (%)
None	81 (89.0%)
Thoracic	2 (2.19%)
Abdominal	4 (4.39%)
No information	4 (4.39%)
Trauma mechanisms	n (%)
Traffic accident	77 (84.61%)
Sports trauma	3 (3.29%)
Fall from height	5 (5.49%)
Ground-level fall	3 (3.29%)
Gunshot wound	2 (2.19%)
Kick from a horse	1 (1.09%)
Nail	n (%)
Antegrade	63 (69.2%)
Retrograde	28 (30.8%)

n = absolute frequencies; % = relative frequencies.

**Table 3:** Postoperative outcomes.

Reoperation	n (%)
Yes	2 (2.2%)
No	89 (97.8%)
Consolidation (Six months)	n (%)
Yes	88 (98.9%)
No	3 (3.3%)
Consolidation (One year)	n (%)
Yes	90 (98.9%)
No	1 (1.1%)
Complications	n (%)
None	88 (96.7%)
Pseudoarthrosis + Infection	1 (1.1%)
Pseudoarthrosis	2 (2.2%)

n = absolute frequencies; % = relative frequencies.

**Table 4:** Contingency table of previous external fixation and fracture type.

		Previous Fixation		
Fracture Type		No	Yes	Total
Close	Observed	76	5	81
	Line %	93.8%	6.2%	100.0%
Open	Observed	5	5	10
	Line %	50.0%	50.0%	100.0%
Total	Observed	81	10	91
	Line %	89.0%	11.0%	100.0%

χ^2^(1) = 17.5, p_Fischer_ < 0.001, power (1-β err prob) = 0.99, actual α = 0.02.

**Table 5:** Contingency table of previous external fixation and consolidation within six month.

Consolidation with Six Months				
Previous Fixation		Yes	No	Total
No	Observed	80	1	81
	Line %	98.8%	1.2%	100.0%
Yes	Observed	8	2	10
	Line %	80.0%	20.0%	100.0%
Total	Observed	88	3	91
	Line %	98.9%	3.3%	100.0%

χ^2^(1) = 8.19, p_Fischer_ = 0.11, power (1-β err prob) = 0.52, actual α = 0.00.

**Table 6:** Contingency table of previous external fixation and consolidation within one year.

Consolidation with One Year				
Previous Fixation		Yes	No	Total
No	Observed	81	0	81
	Line %	100.0%	0.0%	100.0%
Yes	Observed	9	1	10
	Line %	90.0%	10.0%	100.0%
Total	Observed	90	1	91
	Line %	98.9%	1.1%	100.0%

χ^2^(1) = 8.19, p_Fischer_ = 0.11, power (1-β err prob) = 0.88, actual α = 0.00.

**Table 7:** Contingency table of nailing approach and consolidation within six months.

Consolidation with Six Months				
Nailing Approach		Yes	No	Total
Retrograde	Observed	25	3	28
	Line %	89.3%	10.7%	100.0%
Anterograde	Observed	63	0	63
	Line %	100.0%	0.0%	100.0%
Total	Observed	88	3	91
	Line %	96.7%	3.3%	100.0%

χ^2^(1) = 6.98, p_Fischer_ = 0.027, power (1-β err prob) = 0.99, actual α = 0.00.

**Table 8:** Contingency table of nailing approach and consolidation within one.

Consolidation with One Year				
Nailing Approach		Yes	No	Total
Retrograde	Observed	27	1	28
	Line %	96.4%	3.6%	100.0%
Anterograde	Observed	63	0	63
	Line %	100.0%	0.0%	100.0%
Total	Observed	90	1	91
	Line %	98.9%	1.1%	100.0%

χ^2^(1) = 2.27, p_Fischer_ = 0.30, power (1-β err prob) = 0.88, actual α = 0.00.

## Data Availability

The data presented in this study are available from the corresponding author upon reasonable request, in an edited form to exclude sensitive data and personal information, ensuring patient anonymity.

## References

[1] Silva J.C.A., Ribeiro M.D.A., Silva L.N., Pinheiro H.A., Bezerra L.M.A., Oliveira S.B. (2021). Fraturas de Fêmur em Idosos nas Diferentes Regiões do Brasil de 2015 a 2020: Análise dos Custos, Tempo de Internação e Total de Óbitos. Rev. Pesqui. Fisioter..

[2] Lippuner K., Kyuchukova M., Schwab P., Rizzoli R. (2024). Differences in Femoral Fracture Localizations in Men and Women in Switzerland Between 1998 and 2021—Reversal of the Secular Trend?. Osteoporos. Int..

[3] Ghayyad K., Escobar P., Beaudoin T.F., Wandersleben L., Hawks M., Ahmed A., Kachooei A.R. (2024). Nonunion Fractures: Trends in Epidemiology and Treatment of Femur Fractures, 2017–2022. Cureus.

[4] Walter N., Szymski D., Kurtz S.M., Alt V., Lowenberg D.W., Lau E.C., Rupp M. (2023). Femoral Shaft Fractures in Elderly Patients—An Epidemiological Risk Analysis of Incidence, Mortality and Complications. Injury.

[5] Peterle V., Geber J., Darwin W., Lima A., Bezerra P., Novaes M. (2020). Indicators of Morbidity and Mortality by Femur Fractures in Older People: A Decade-Long Study in Brazilian Hospitals. Acta Ortop. Bras..

[6] Te D., Fe D. (2022). Diaphyseal Femoral Fracture Fixation: Outcome of 108 Consecutive Fixations in a Tertiary Hospital. Sch. J. Appl. Med. Sci..

[7] Haase D.R.M., Saiz A.M.J., Eastman J.G., Achor T.S., Choo A.M., Munz J.W., Warner S.J. (2024). Ipsilateral Femoral Neck and Shaft Fractures: Complex Injuries with High Rates of Femoral Shaft Nonunion. J. Orthop. Trauma.

[8] Xie W., Liu H., Chen S., Xu W., Lin W., Chen T., Zhu L., Zhai W., Wu J. (2024). Comparison of Three Internal Fixation Constructs for AO/OTA 33-A3 Distal Femoral Fractures: A Biomechanical Study. Bioengineering.

[9] Vasilopoulou A., Karampitianis S., Chloros G.D., Giannoudis P.V. (2024). Incidence of Complications and Functional Outcomes Following Segmental Femoral Shaft Fractures: A Critical Review of the Literature. Eur. J. Orthop. Surg. Traumatol..

[10] Enninghorst N., McDougall D., Evans J.A., Sisak K., Balogh Z.J. (2013). Population-based epidemiology of femur shaft fractures. J. Trauma Acute Care Surg..

[11] Hosseini H., Heydari S., Domari A.A., Raesi R., Hushmandi K., Faryabi R., Gharaee M., Daneshi S. (2024). Comparison of Treatment Results of Femoral Shaft Fracture with Two Methods of Intramedullary Nail (IMN) and Plate. BMC Surg..

[12] Wu K.J., Li S.H., Yeh K.T., Chen I.H., Lee R.P., Yu T.C., Peng C.H., Liu K.L., Yao T.K., Wang J.H. (2019). The Risk Factors of Nonunion After Intramedullary Nailing Fixation of Femur Shaft Fracture in Middle-Aged Patients. Medicine.

[13] Ricci W.M., Gallagher B., Haidukewych G.J. (2009). Intramedullary Nailing of Femur Shaft Fractures: Current Concepts. J. Am. Acad. Orthop. Surg..

[14] Brinker M.R., O’Connor D.P. (2007). Exchange Nailing of Ununited Fractures. J. Bone Joint Surg. Am..

[15] Perisano C., Cianni L., Polichetti C., Cannella A., Mosca M., Caravelli S., Maccauro G., Greco T. (2022). Plate Augmentation in Aseptic Femoral Shaft Nonunion after Intramedullary Nailing: A Literature Review. Bioengineering.

[16] Louka J.G., Seligson D., Vig K.S., Khushdeep S.V., Zamora R., Zou J., Carlson J.B., Daccarett M. (2025). Femoral shaft fracture with a third fragment treated with an intramedullary nail: Is the displacement of the third fragment predictive of nonunion?. Eur. J. Orthop. Surg. Traumatol..

[17] Ghouri S.I., Mustafa F., Kanbar A., Al Jogol H., Shunni A., Almadani A., Abdurraheim N., Goel A.P., Abdelrahman H., Babikir E. (2023). Management of Traumatic Femur Fractures: A Focus on the Time to Intramedullary Nailing and Clinical Outcomes. Diagnostics.

[18] Kang N.W.W., Tan W.P.J., Phua Y.M.C., Min A.T.G., Naidu K., Umapathysivam K., Smitham P.J. (2021). Intramedullary Nail: The Past, Present and the Future—A Review Exploring Where the Future May Lead Us. Orthop. Rev..

[19] Koso R.E., Terhoeve C., Steen R.G., Zura R. (2018). Healing, Nonunion, and Re-Operation After Internal Fixation of Diaphyseal and Distal Femoral Fractures: A Systematic Review and Meta-Analysis. Int. Orthop..

[20] Tsang S.T.J., Mills L.A., Baren J., Frantzias J., Keating J.F., Simpson A.H.R.W. (2015). Exchange Nailing for Femoral Diaphyseal Fracture Non-Unions: Risk Factors for Failure. Injury.

[21] Saleeb H., Tosounidis T., Papakostidis C., Giannoudis P. (2019). Incidence of Deep Infection, Union, and Malunion for Open Diaphyseal Femoral Shaft Fractures Treated with IM Nailing: A Systematic Review. Surgeon.

[22] Davidson A., Houri S.S., Cohen J., Feldman G., Mosheiff R., Liebergall M., Weil Y.A. (2022). Initial Definitive Treatment of Open Femoral Shaft Fractures with Retrograde Nailing—Is It Safe? A Retrospective Analysis Comparing Antegrade to Retrograde Nailing. Injury.

[23] Pires R.E.S., Fernandes H.J.A., Belloti J.C., Balbachevsky D., Faloppa F., dos Reis F.B. (2006). Como são tratadas as fraturas diafisárias fechadas do fêmur no Brasil? Estudo transversal. Acta Ortop. Bras..

[24] Vasconcelos P.A.B., Rocha A.J., Fonseca R.J.S., Teixeira T.R.G., Mattos E.S.R., Guedes A. (2020). Femoral fractures in the elderly in Brasil—incidence, lethality, and costs (2008–2018). Rev. Assoc. Med. Bras..

[25] Cobra C.R.M.N., Garcia P.C., Passos I.C.M.O., Rocha G.D.S., Nogueira L.S. (2024). Analysis of intensive care unit admissions for older adults with femoral fractures: A retrospective cohort. Rev. Esc. Enferm. USP.

[26] Trincado R.M., Mori M.A.K., Fernandes L.S., Perlaky T.A., Hungria J.O.S. (2022). Epidemiology of proximal femur fracture in older adults in a philanthropical hospital in São Paulo. Acta Ortop. Bras..

[27] El Fatah S.A., Nunes W.F., Katz M., Queiroz H.R., Fontana J.K.K., Ikeda R.E. (2022). Epidemiological profile of proximal femoral fractures in older adults at the regional hospital in Cotia—SP, Brazil. Acta Ortop. Bras..

[28] Tischler E.H., McDermott J.R., Wolfert A.J., Krasnyanskiy B., Ibrahim I., Malik A.N., Gross J.M., Suneja N. (2023). Predictors of 30-day mortality, unplanned related readmission and reoperation among isolated closed femoral shaft fractures. J. Orthop..

[29] Oliveira P.R., Leonhardt M.C., Carvalho V.C., Kojima K.E., Silva J.S., Rossi F., Lima A.L.L. (2018). Incidence and risk factors associated with infection after intramedullary nailing of femoral and tibial diaphyseal fractures: Prospective study. Injury.

[30] Fu H., Hu L., Zou F., Liao X., Zheng Y., Jin P., Jia J., Xu J. (2024). A Comparative Study of the Early Postoperative Outcome of Three Intramedullary Fixation Modalities in the Treatment of Intertrochanteric Fractures of the Femur in the Elderly. J. Musculoskelet Neuronal Interact.

[31] de Moraes F.B., da Silva L.L., Ferreira F.V., Ferro A.M., da Rocha V.L., Teixeira K.I. (2015). Epidemiological and radiological evaluation of femoral shaft fractures: Study of 200 cases. Rev. Bras. Ortop..

[32] Viel I.L., Moura B.R.S., Martuchi S.D., Nogueira L.d.S. (2019). Factors Associated With Interhospital Transfer of Trauma Victims. J. Trauma Nurs..

[33] Puccetti V.L.Y.A., de Miranda F.L., de Figueiredo C.C.N., Medeiros K.A.A., Leonhardt M.C., Silva J.D.S., Kojima K.E. (2024). Risk Factors at Non-Union of Tibial Fracture Treated with Intramedullary Nail. Acta Ortop. Bras..

[34] Basile G., Fozzato S., Petrucci Q.A., Gallina M., Prevot L.B., Accetta R., Zaami S. (2022). Treatment of Femoral Shaft Pseudarthrosis: Case Series and Medico-Legal Implications. J. Clin. Med..

[35] Marín S., Martínez F., Such F., Ripoll J., Beltrán O., Munuera M., López J., Campos J. (2021). Risk factors for high length of hospital stay and in-hospital mortality in hip fractures in the elderly. Rev. Esp. Cir. Ortop. Traumatol..

[36] Cho W., Hwang T., Choi Y., Yang J., Kim M., Jo S., Cho W., Oh S. (2019). Diastolic dysfunction and acute kidney injury in elderly patients with femoral neck fracture. Kidney Res. Clin. Pract..

[37] Souza A., Thaffarell P., Freitas G.P., Lopes H.B., Weffort D., Adolpho L.F., Gomes M.P.O., Oliveira F.S., Almeida A.L.G., Beloti M.M. (2024). Efficacy of mesenchymal stem cell-based therapy on the bone repair of hypertensive rats. Oral Dis..

[38] Mills L.A., Aitken S.A., Simpson A. (2017). The Risk of Non-Union per Fracture: Current Myths and Revised Figures from a Population of Over 4 Million Adults. Acta Orthop..

[39] Hoffmann M.F., Khoriaty J.D., Sietsema D.L., Jones C.B. (2019). Outcome of intramedullary nailing treatment for intertrochanteric femoral fractures. J. Orthop. Surg. Res..

[40] Della Rocca G.J., Crist B.D. (2006). External Fixation Versus Conversion to Intramedullary Nailing for Definitive Management of Closed Fractures of the Femoral and Tibial Shaft. J. Am. Acad. Orthop. Surg..

[41] Zhang F., Zhu L., Li Y., Chen A. (2015). Retrograde Versus Antegrade Intramedullary Nailing for Femoral Fractures: A Meta-Analysis of Randomized Controlled Trials. Curr. Med. Res. Opin..

